# Exosomal miR-17-5p, miR-146a-3p, and miR-223-3p Correlate with Radiologic Sequelae in Survivors of COVID-19-Related Acute Respiratory Distress Syndrome

**DOI:** 10.3390/ijms241713037

**Published:** 2023-08-22

**Authors:** Rosa Curcio, Giulia Poli, Consuelo Fabi, Chiara Sugoni, Maria Bruna Pasticci, Roberto Ferranti, Monica Rossi, Ilenia Folletti, Leandro Sanesi, Edoardo Santoni, Irene Dominioni, Massimiliano Cavallo, Giovanni Morgana, Lorenzo Mordeglia, Giovanni Luca, Giacomo Pucci, Stefano Brancorsini, Gaetano Vaudo

**Affiliations:** 1 Unit of Internal Medicine, Santa Maria Terni Hospital, 05100 Terni, Italy; 2 Department of Medicine and Surgery, University of Perugia, 06132 Perugia, Italy; 3 Infectious Diseases Unit, Santa Maria Terni Hospital, 05100 Terni, Italy; 4 Unit of Radiology, Santa Maria Terni Hospital, 05100 Terni, Italy; 5 Section of Occupational Medicine, Santa Maria Terni Hospital, 05100 Terni, Italy

**Keywords:** COVID-19, miRNA, NLRP3 inflammasome, fibrosis, respiratory failure, high-resolution computed tomography

## Abstract

We investigated the association between circulating microRNAs (miRNAs) potentially involved in the lung inflammatory process and fibrosis development among COVID-19-related acute respiratory distress syndrome (ARDS) survivors. At 4 ± 2 months from clinical recovery, COVID-19-related ARDS survivors matched for age, sex, and clinical characteristics underwent chest high-resolution computerized tomography (HRCT) and were selected based on imaging pattern evolution into fully recovered (N = normal), pulmonary opacities (PO) and fibrosis-like lesions (FL). Based on the previous literature, we performed plasma miRNA profiling of exosomal miRNAs belonging to the NLRP3-inflammasome platform with validated (miR-17-5p, miR-223-3p) and putative targets (miR-146a-5p), miRNAs involved in the post-transcriptional regulation of acute phase cytokines (miR128-3p, miR3168, miR125b-2-3p, miR106a-5p), miRNAs belonging to the NLRP4-inflammasome platform (miR-141-3p) and miRNAs related to post-transcriptional regulation of the fibrosis process (miR-21-5p). miR-17-5p, miR-223-3p, and miR-146a-5p were significantly down-regulated in patients with FL when compared to patients with PO. miR-146a-5p was also down-regulated in patients with FL than in N. The expression of the remaining miRNAs did not differ by group. In patients with long-term pulmonary radiological sequelae following COVID-19-related ARDS, a down-regulation of miR-17-5p, miR-146a-3p, and miR-223-3p correlated to fibrosis development in patients showing persistent hyper-reactivity to inflammatory stimulation. Our results support the hypothesis that NLRP3-Inflammasome could be implicated in the process of fibrotic evolution of COVID-19-associated ARDS.

## 1. Introduction

Since December 2019, SARS-CoV-2 and the related COVID-19 affected over 760 million people and was responsible for nearly seven million deaths worldwide [1]. Despite global vaccination led to a drastic decrease in the number of deaths and hospitalizations [2], many infected patients faced incomplete recovery of health status and with persistence of long-term symptoms [3]. Impaired pulmonary function and physical performance were often found after several months of clinical recovery in people with COVID-19 and the degree of impairment correlated with the severity of the acute disease [4]. Approximately a third of patients surviving critical COVID-19 also showed long-term residual typical radiological abnormalities at chest high-resolution computed tomography (HRCT) [5,6]. Moreover, some radiological features such as reticulations and traction bronchiectasis [7] were found to persist or even increase at 1 year, raising the question of whether the evolution towards a pulmonary fibrosis-like pattern is related to irreversible lung dysfunction and residual functional disability.

It is generally thought that fibrotic remodeling is the result of an abnormal wound-healing process. Such a process is primed by the persistence of a sub-acute inflammatory status, despite apparent clinical recovery [8]. Dysregulation of the immune response is also implicated in tissue damage, extracellular matrix deposition, and interstitial fibrosis [9,10]. However, little is known about the mechanisms regulating the inter-individual predisposition to persistent inflammation and development of fibrosis following acute lung damage in critical COVID-19.

MicroRNAs (miRNAs) are short non-coding miRNAs mediating the post-transcriptional repression or mRNA degradation according to epigenetic mechanisms [11,12]. The microRNA (miRNA)-guided RNA silencing pathway is a gene regulatory process that is involved in the repressive control of messenger RNA (mRNA) translation. Exosomal miRNA are enveloped into 50–150 nm endosome-derived extracellular vesicles with other biological molecules, such as proteins, DNAs, and lipids [13] which are secreted by various cell types widely distributed in many bodily fluids such as blood, urine, ascitic and amniotic fluid. Exosomal miRNAs play an important role in the exchange of biological information between different cells [14] and are protected from RNase degradation [15]. However, little is known about the potential role of exosomal miRNAs in the long-term persistence of pulmonary inflammation and fibrotic evolution of acute lesions.

Several exosomal miRNAs, such as miR-17-5p, miR-146a-5p, miR-223-3p, miR106a-5p, and miR-141-3p are known to exert a post-transcriptional regulation on NLRs-inflammasome pathway. Their hyperactivation has been hypothesized to play a role in inflammation progression and cytokine storm in acute COVID-19 [16,17]. Similarly, other circulating miRNAs were found to be associated with diffusing capacity and radiological features in patients developing COVID-19-related lung disease after three months from discharge as compared to controls, suggesting possible molecular pathways underlying the pathogenesis of pulmonary sequelae in COVID-19 patients [18]. Other miRNAs, such as those involved in the post-transcriptional regulation of acute phase cytokines (miR128-3p and miR100-5p) [19,20], and those found to be associated with fibrotic lung disease (miR-21-5p and miR210-3p) might also be hypothesized to play a role in lung disease persistence and progression [21].

The aim of the present study was to correlate the expression of exosomal miRNAs to different radiological patterns observed in a cohort of subjects who survived COVID-19-related acute respiratory distress syndrome (ARDS) after several months from clinical recovery. Subjects were classified according to HRCT findings into those with almost complete lung recovery and those with persistence of residual pulmonary opacities; the latter were further classified according to the presence/absence of signs of fibrotic evolution of lung lesions.

## 2. Results

### 2.1. Characteristics of the Study Population

A total of 31 patients were evaluated: 11 patients with normal HRCT at follow-up, corresponding to a pulmonary opacities (PO) score < 3 and fibrotic-like lesions (FL) score < 3 (N pattern), 11 patients with PO score ≥ 3 and FL score < 3 at follow-up (PO pattern), and 9 patients with FL score ≥ 3 at follow-up (FL pattern). Clinical characteristics of the total study population and by groups are presented in Table 1.

The three groups did not show significant differences in terms of age and sex distribution. Although not significant, some differences were noted between groups: patients with the N pattern were more frequently current smokers and had a relatively higher rate of patients with type 2 diabetes mellitus. Patients with FL and PO patterns had more often a positive history of previous cardiovascular disease and chronic obstructive pulmonary disease (COPD). The average length of stay was slightly longer for patients with FL patterns compared to the N pattern, although the difference was not statistically significant. Patients with the FL pattern showed a not significantly slightly lower peak oxygen consumption than other groups; no differences between groups were observed in terms of both serum c-reactive protein (CRP) and ferritin levels during the acute phase of the disease and at follow-up.

### 2.2. Radiological Findings

Radiological findings during the acute phase and at follow-up are displayed in Table 2.

On average, patients with the FL pattern showed the highest lobe extension of PO during the acute phase, although the difference between the three groups was not significant (FL: 16.0 ± 6. PO: 13.6 ± 4, N: 12.6 ± 4, range 0–25, *p* = 0.21). By contrast, whereas patients with the N pattern showed almost complete regression of ground-glass opacities (GGO) at follow-up and a minimal extension of reticulation pattern (0.2 ± 1), both patients with the FL and PO patterns showed persistence of GGO and consolidations at follow-up, despite lobe involvement was significantly reduced as compared to the acute phase (FL: 4.6 ± 1. PO: 3.9 ± 1, *p* between groups: 0.27). FL, such as reticulations and traction bronchiectasis, were marginally observed also in patients with PO pattern (0.6 ± 1) and N pattern (0.2 ± 1, *p* between groups 0.29).

### 2.3. miRNAs

**Figure 1:**
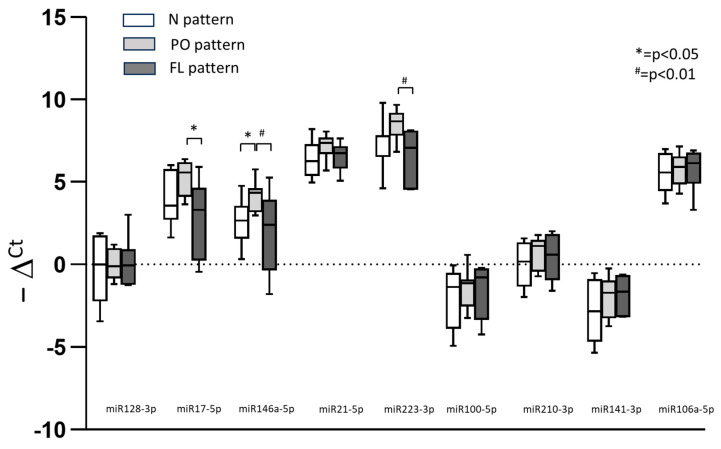
Expression levels of tested miRNAs in subjects with high-resolution CT scan findings suggestive of fully recovering from pulmonary disease (N pattern), the persistence of pulmonary opacities (PO pattern), and the presence of fibrosis-like lesions (FL pattern) at follow-up, * *p* < 0.05, # *p* < 0.01. Data are expressed as −ΔCt.

In subjects presenting the FL pattern at follow-up, miR-17-5p, miR-223-3p, and miR-146a-5p were found to be significantly down-regulated compared to subjects presenting the PO pattern (Figure 1). The miR-146a-5p was found to be significantly under-expressed in subjects who fully recovered from pulmonary radiological signs of disease (N pattern) as compared to those with PO pattern (*p* < 0.05). None of the other tested miRNAs (miR128-3p, miR21-5p, miR100-5p, miR210-3p, miR141-3p, and miR106a-5p) showed significant differences between groups.

## 3. Discussion

Our study revealed that miR-17-5p, miR-223-3p, and miR-146a-5p were significantly down-regulated in patients with HRCT findings suggestive of pulmonary opacities with fibrosis-like evolution at 4 ± 2 months after clinical recovery for COVID-19-related ARDS, as compared with subjects showing only persistence of PO, such as ground-glass opacities and consolidations. Notably, these two groups did not differ in terms of age, sex, main clinical characteristics, and extension of radiological pulmonary opacities at follow-up. We also showed that in patients with PO pattern, miR-146a-5p was significantly over-expressed as compared with subjects who fully recovered from ARDS at follow-up. Given that miR-17-5p, miR-146a-5p, and miR-223-3p have been reported in previous literature as validated and putative NLRP3 inflammasome-related exosomal miRNAs, our results are in keeping with the hypothesis that the dysregulation of the NLRP3-inflammasome pathway could play a role in promoting fibrotic evolution in post-acute severe COVID-19 disease.

The analysis of morpho-phenotypical characteristics of patients with residual parenchymal lung disease over the long term after critical COVID-19 has been the object of extensive research. It is known that lung involvement could display distinct patterns. Ravaglia and colleagues [22] identified three possible clusters in post-acute COVID-19 symptomatic patients, one characterized by radiologic lung fibrotic appearances of architectural distortion and traction bronchiectasis corresponding to fibrosing interstitial pneumonia, one characterized by consolidations, ground glass peri-lobular pattern and inflammatory infiltrate, and one characterized by mild residual lung disease with minor vascular abnormalities. The underlying pathogenetic mechanism remains still unknown, although specific pathways involved in promoting inflammation and immune derangement have been evoked.

The dysregulation of the NLRP3-inflammasome pathway has been involved in the pathogenesis of various pulmonary diseases characterized by fibrotic evolution such as acute lung injury, respiratory infections, chronic obstructive pulmonary disease, asthma [23], pulmonary hypertension [24], cystic fibrosis, and idiopathic pulmonary fibrosis [25]. Recently, the hyperactivation of NLRP3 inflammasome followed by IL-1β was indicated as a potential regulatory factor in the pathogenesis of COVID-19, similar to other inflammatory diseases [17,26].

miR-146a was described to play a role in attenuating the response to an inflammatory stimulus. Indeed, it suppresses NLRP3- inflammasome activation in macrophages by reducing protein levels of NLRP3/ASC/caspase1 [27], and its expression, induced by NF-κB, was also associated with inhibition of IL-1 and TNF-α receptors synthesis [28]. A downregulation of miR-146a-5p could therefore promote the persistence of an inflammatory process [29,30,31]. miR-146a-5p expression was also negatively correlated with target mRNAs IL-1 receptor-associated kinase 1 (IRAK1), IRAK2, and tumor necrosis factor receptor associated-factor 6 (TRAF6), which participate in the nuclear factor-κB (NF-κB) pro-inflammatory signaling pathway [32,33]. Of note, a miR-146 deficiency in diabetes correlates with increased inflammation (for insufficient inhibition of IRAK1/TRAF6) and fibrosis (for expression of fibronectin) [34]. Lambert and colleagues [35] proposed a model in which miR-146a is induced by the inflammatory stimulus as negative feedback to limit inflammation itself; exogenous administration of miR-146a would increase the effect of glucocorticoids. miR-146a-5p was also described to regulate inflammation during COVID-19. Indeed, Sabbatinelli showed that COVID-19 patients who did not respond to tocilizumab had lower serum levels of miR-146a-5p after the treatment; among non-responders, those with the lowest serum levels of miR-146a-5p experienced the most adverse outcome [36]. MiR-146a-5p has been described as a key modulator of the inflammatory response to acute lung injury, such as that induced by sulfur mustard. In mouse models, miR-146a-5p-overexpressing extracellular vesicles (miR-146a-5p+-EVs) or miR-146a-5p-underexpressing extracellular vesicles were found to significantly up- or downregulate the inflammatory lung reaction [37]. ARDS could be viewed as the most severe form of acute lung injury and, accordingly, Roganovic hypothesized that the downregulation of circulating miR-146 in people with diabetes, obesity, and hypertension could be reflected by enhanced inflammation and fibrosis, as systemic effects accompanying severe COVID-19 [34].

miR-223 is another regulator of NLRP3 transcription. In murine macrophages, miR-223 negatively interacts with NLRP3 3′-UTR mRNA [38]. Granulocytes without miR-223 are hypermature and hypersensitive to activating stimuli. As a result, mutant miR-223 mice spontaneously develop inflammatory pulmonary pathology and respond with exaggerated destruction of the tissue to the stimulus of the endotoxin [39]. Studies focusing on miR-223 target genes showed that PARP-1 suppression by miR-223 is involved in the resolution of pneumonia, in that miR-223 deficiency was associated with severe lung inflammation, whereas pulmonary overexpression of miR-223 in mice resulted in protection during acute lung injury-induced by mechanical ventilation or by infection [40]. The role of miR-223 in hematopoietic line-age differentiation and inflammatory response has also been investigated [41]. Recent studies identified a differential expression of miR-223-3p in patients with pulmonary inflammation or respiratory syndromes. In vivo studies reported that COVID-19 mice with inhibited miRNA-223-3p present an over-expression of anti-inflammatory factors and reduced pulmonary inflammation. Furthermore, expression analysis also revealed NLRP3 mRNA increased levels after miRNA-223-3p inhibition [42]. These results suggested that miRNA-223 actively participates in the regulation of lung inflammatory response induced by SARS-CoV-2, as it was proposed by some authors [16,17].

The miR-17-5p is a member of the miR-17~92 cluster and inhibits the activation of NLRP3. Dakhlallah and co-workers [43], in their study, showed that the introduction of the miR-17~92 cluster in lung fibroblasts with idiopathic fibrosis reduced fibrosis and normalized cellular phenotype by reducing DNA methylation of the cluster. Recently, miR-17-5p, along with other miRNAs, was found to be associated with COVID-19 pulmonary sequelae in patients with vs. without ARDS [18].

Although the possibility that the level of miRNA expression only reflects the current degree of inflammation in the lung interstitial tissues cannot be ruled out, our results could be viewed as in agreement with the hypothesis that the epigenetic regulation of NLRP3 inflammasome could have a role in promoting lung persistent inflammation and fibrotic transition in subjects with post-acute lung sequelae of severe COVID-19. Further research is therefore needed to determine the plasma levels of proteins whose transcription is regulated by these miRNAs, as well as to evaluate knockout rats for the genes coding for related miRNAs.

Overall, the present results show that in patients with persistent radiological features of the disease and thus with a persistent inflammatory stimulus at four months after the acute phase of COVID-19-related ARDS, a down-regulation of NLRP3-inflammasome-related miRNAs, such as miR-17-5p, miR-146a-3p, and miR223-3p could predispose to the development of the fibrosis-like radiological pattern. Our results, therefore, suggest that NLRP3-inflammasome-related miRNAs and their regulated cytokines or receptors could represent a novel diagnostic or therapeutic target in pulmonary sequelae of COVID-19-related ARDS.

Our results should be viewed in light of their limitations, including the previously cited need for further studies to evaluate the epigenetic contribution to the regulation of NLRP3 inflammasome and to analyze the expression of proteins whose transcription could be regulated by the miRNAs under evaluation. One of the main limitations of this study was the lack of specificity of miR-17-5p, miR-146a-3p, and miR-223-3p for fibrotic lung disease, which could implicate a lack of applicability in clinical practice. However, in light of the previous literature, there is a plausible basis and sufficient rationale to consider these miRNAs as potentially involved in the modulation of lung inflammation and fibrotic evolution in the post-acute phase of COVID-19-associated lung disease. Given the small sample size, another limitation is that our result could only generate hypotheses, and results should be confirmed in larger datasets. Furthermore, several risk factors could influence the expression of peripheral ncRNAs. Previous research has indicated that almost all cardiovascular risk factors could result in different expression of some miRNAs in peripheral blood, including age [44], obesity [45], and type 2 diabetes [46]. Despite all the attempts to select well-balanced groups of patients with similar clinical features, we could not exclude that inherent differences in other clinical characteristics between groups could have influenced miRNA expression. We could not rule out that PO could have masked the presence of fibrosis-like lesions, leading to wrong patient classification. For this reason, a longer follow-up with longitudinal HRCTs is needed to confirm the results. Finally, our results do not provide sufficient evidence to consider the expression of exosomal miR-17-5p, miR-146a-3p, and miR-223-3p as selective regulating factors of NLRP3 during the post-acute phase of COVID-19. Such a hypothesis needs further confirmation in dedicated studies.

## 4. Materials and Methods

All survivors of COVID-19-related pneumonia and ARDS, who were initially admitted to the “Santa Maria” University Hospital (Terni) during the period between December 2020 and April 2021, were proposed to participate in an observational prospective registry named “CArdioPulmonary Exercise Testing for a global fitness Assessment in patients with recent COVID-19 Interstitial Pneumonia (CAPTAIN) study”. The registry aimed to evaluate and quantify the long-term sequelae of COVID-19-related respiratory failure. A diagnosis of acute COVID-19 was based on the positivity of viral RNA at the RT-PCR on the nasopharyngeal swab performed at hospital admission, according to standardized procedures [47]. The same test was repeated at a follow-up visit and was negative for all participants. COVID-19-associated ARDS was diagnosed based on the Berlin definition [48] according to radiological and clinical findings at Hospital admission.

Participation was proposed to all subjects aged >18 years old and able to sign a written informed consent. The study protocol was approved by the Local Ethics Committee (protocol number 50970). The research was carried out according to The Code of Ethics of the World Medical Association (Declaration of Helsinki). All patients were proposed to undergo a follow-up visit after on average 4 ± 2 months from hospital discharge, consisting of a clinical and instrumental evaluation including blood sample testing, a HRCT scan, and a cardiopulmonary exercise testing (CPET). Patients were excluded from CPET evaluation if they were aged >75 years, or if they had a history of heart failure with ejection fraction < 50%, symptomatic coronary heart disease, history of moderate/severe valvulopathy, atrial fibrillation, asthma or severe chronic obstructive pulmonary disease, or musculoskeletal disease affecting the physical performance.

HRCT and image analysis were performed in the hospital premises by two radiologists (RF and MR) with more than 5 years of experience in chest imaging, both during the acute disease and at follow-up. Lung sequelae were classified according to quantitative scores based on the degree of each lobe involvement (0 = none, 1 = <5%, 2 = 5–25%, 3 = 26–50%, 4 = 51–75%, 5 > 75%, range 0–25) and to qualitative scores based on radiological features of PO, including GGO, linear opacities, crazy-paving and consolidations, and fibrosis-like lesions (FL) including reticulation, traction bronchiectasis and honeycombing [49,50]. An agreement was reached by consultation in case of discrepancies.

**Figure 2:**
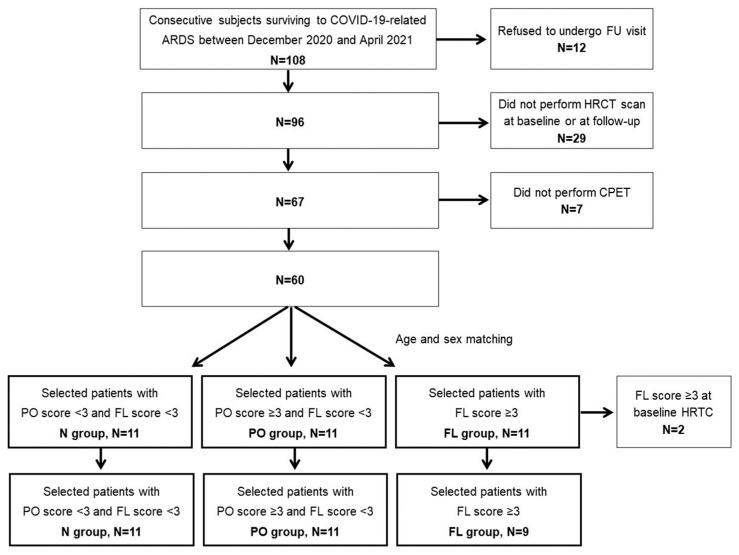
Flowchart of the patient selection for the study.

For the present study, from an initial cohort of 108 patients, those who refused to undergo the follow-up visit (n = 12), who did not perform an HRCT scan during the acute phase or at follow-up (n = 29), and those who refused to perform CPET (n = 7), were excluded. Among the remaining 60 patients, three age- and sex-matched groups of 11 patients each was selected among those with a quantitative score ≥ 3 for PO and no signs of FL (PO pattern), those with a quantitative score ≥ 3 for FL (FL pattern), and those with a quantitative score < 3 for both PO and FL (N pattern). Patients with FL at baseline HRCT (n = 2) were also excluded from selection. The patient selection process was summarized in Figure 2.

Blood samples of selected patients were collected in EDTA tubes from a peripheral vein and kept immediately on ice. Plasma was separated by centrifugation (4000 rpm for 15 min at 4 °C), aliquoted and analyzed. Based on the previous literature [16,17], we performed plasma miRNA profiling of the following exosomal miRNAs: exosomal miRNAs belonging to the NLRP3-inflammasome platform with validated (miR-17-5p, miR-223-3p) and putative targets (miR-146a-5p), miRNAs involved in the post-transcriptional regulation of acute phase cytokines (miR128-3p, miR3168, miR125b-2-3p, miR106a-5p), miRNAs belonging to the NLRP4-inflammasome platform (miR-141-3p) and miRNAs related to post-transcriptional regulation of the fibrosis process (miR-21-5p). The list of miRNA evaluated in the present study and the related mRNA targets are shown in Table 3.

Exosome precipitation, isolation, and miRNA extraction were performed with “Total Exosomal RNA, Including miRNA, from Serum and Plasma” (Qiagen, Gernantown, MD, USA), according to the manufacturer’s instructions. One volume of plasma was mixed with Buffer XBP. Sample/XBP mix was added to the exoEasy spin column and spun for 1 min at 500× *g*. Flow-through was discarded. A 3.5 mL of Buffer XWP were added to the exoEasy Midi spin column and centrifugated for 5 min at 5000× *g*. The spin column was transferred to a fresh collection tube and 700 μL of QIAzol was added to the membrane. After a spin for 5 min at 5000× *g*, the lysate was collected. At this point, spike-in controls were added to the lysate and mixed with 90 μL of chloroform. Through mixing, a phase separation was obtained. After centrifugation for 15 min at 12,000× *g* at 4 °C, the sample was separated into 3 phases: an upper, colourless aqueous phase containing RNA, a thin, white interphase, and a lower, red, organic phase. A 400 μL of the upper phase were transferred to a new collection tube. 2 volumes of ethanol were added and 700 μL of the sample were replaced into an RNeasy MinEluate spin column. A 700 μL Buffer RWT was mixed with the sample and after centrifugation for 15 s at 8000× *g*, the flow-through was discarded. A 500 μL Buffer RPE onto the RNeasy MinEluate spin column was pipetted and after centrifugation for 2 min at 8000× *g*, the RNeasy MinEluate spin column was placed in a new 1.5 mL collection tube. A 14 μL of RNase-free water was added directly to the center of the spin column membrane. After a spin for 1 min at full speed, RNA including miRNA was obtained. A 30 ng of RNA diluted with nuclease-free water were reverse transcribed with cDNA miRCURY LNA™ RT kit (Qiagen, Gernantown, MD, USA). The total reaction volume was 10 μL (2 μL of Reaction Buffer, 1 μL of Enzyme Mix, 0.5 μL of RNA spike-incontrol UniSp6 as a positive cDNA synthesis control). Samples were kept at 42 °C for 60 min, and cDNA was immediately used for real-time PCR assays with miRCURY LNA™ SYBR^®^ Green PCR kit (Qiagen, Gernantown, MD, USA). Stratagene Mx3005XP instrument (Agilent Technology, Santa Clara, CA, USA) was used for PCR assay. A 2 μL of cDNA diluted 1:30 with nuclease-free water was used in a total reaction volume of 10 μL. Melting curve analysis was carried out, each sample was run in triplicate, and results were averaged; no template controls were included in the analysis. RNU6 was used to normalize data. The −ΔCt method was used to calculate the relative expression of the target genes as follows: −ΔCt miRNA= −(Cttarget−CtRNU6).

### Statistical Analysis

Continuous variables were reported as mean ± standard deviation (SD) if normally distributed or as median [interquartile range (IQR)] if non-normally distributed. The assumption of satisfactory normal distribution was tested for all the examined variables by the Kolmogorov–Smirnov Z test. Binary variables were summarized using frequency and percentage and compared using χ2/Fisher exact test. Differences between groups were compared by the one-way analysis of variance (ANOVA), with a further post-hoc affirmation of Tukey’s honestly significant difference test to detect the significance of pairwise comparisons. The Kruskal–Wallis test was used to compare means between independent samples if the homogeneity of variances was not satisfied. All *p*-values were 2-tailed, and the level of significance was set at *p* ≤ 0.05. Statistical analyses were performed using version 22.0 of IBM SPSS (SPSS, Inc., Chicago, IL, USA) and GraphPad Prism 6.0 (GraphPad Software, San Diego, CA, USA).

## 5. Conclusions

The miR-17-5p, miR-146a-3p, and miR-223-3p are correlated to fibrosis development in patients showing persistent hyper-reactivity to inflammatory stimulation during the post-acute phase of COVID-19-related RDS. have been reported in the previous literature as validated and putative NLRP3 inflammasome-related exosomal miRNAs, our results are in keeping with the hypothesis that the dysregulation of the NLRP3-inflammasome pathway could play a role in promoting fibrotic evolution in post-acute severe COVID-19 disease. The study of the individual expression of inflammasome-related miRNAs may help to modulate the therapy on each patient rather than on each disease. Further insight into the molecular bases of fibrotic evolution of diseases induced by inflammatory responses to external factors such as SARS-CoV-2 infection is needed.

## Figures and Tables

**Table 1:** Clinical characteristics of the study population divided by HRCT patterns at follow-up.

	Total	Fibrotic-like Lesions (FL)	Pulmonary Opacities (PO)	Normal (N)	*p*
	N = 31	N = 9	N = 11	N = 11	
Age, years	60 ± 6	61 ± 6	60 ± 6	58 ± 7	0.25
Male sex, %	33	44	36	33	0.42
Height, cm	170 ± 7	168 ± 9	171 ± 11	171 ± 7	0.55
BMI, Kg/m^2^	31.1 ± 6.6	31.7 ± 7.2	30.1 ± 5.7	30.7 ± 6.6	0.56
Current smokers, %	7	0	0	18	0.21
Hypertension, %	61	54	60	71	0.79
T2DM, %	10	0	9	20	0.44
COPD, %	10	12	16	0	0.41
Previous CV event, %	4	14	0	0	0.25
Hospital length of stay, days	18 ± 9	23 ± 10	18 ± 8	15 ± 6	0.13
Serum lymphocytes, mm^3^	791 ± 351	833 ± 377	692 ± 391	859 ± 297	0.51
CRP acute phase, mg/dL	6.9 [4.0–12.0]	7.5 [2.5–12.5]	6.5 [4.2–16.8]	6.1 [3.3–10.6]	0.88
Ferritin acute phase, ng/mL	950 [426–1300]	804 [534–1579]	1205 [674–1331]	400 [359–1133]	0.45
ETI, %	6	11	0	9	0.52
NIV, %	36	44	38	27	0.75
cPAP, %	12	11	15	9	0.90
Time from discharge, days	121 ± 39	118 ± 41	139 ± 43	105 ± 18	0.16
Dyspnea, %	27	33	8	45	0.11
Asthenia, %	6	11	0	9	0.51
Depression, %	18	22	15	18	0.92
Peak oxygen consumption, mL/min/Kg	18.4 ± 5	18.9 ± 4	18.4 ± 4	17.8 ± 5	0.80
CRP follow-up, mg/dL	0.2 [0.1–0.3]	0.1 [0.1–0.4]	0.2 [0.1–0.5]	0.2 [0.1–0.3]	0.61
Ferritin follow-up, ng/mL	108 [34–245]	126 [42–215]	138 [29–293]	62 [26–214]	0.43

Data are shown as mean ± SD or as median [IQR]. BMI: body mass index; T2DM: type 2 diabetes mellitus; COPD: chronic obstructive pulmonary disease; CV: cardiovascular; CRP: c-reactive protein; ETI: endotracheal intubation; NIV: non-invasive ventilation; cPAP: continuous positive airway pressure.

**Table 2:** Radiological findings of the study population during the acute phase of COVID-19-related ARDS and at follow-up. PO pattern included ground-glass opacities and consolidations. FL pattern included reticulations and traction bronchiectasis.

	Fibrotic-like Lesions (FL)	Pulmonary Opacities (PO)	Normal (N)	*p* (ANOVA)
GGO, acute phase	13.1 ± 4	9.8 ± 3	8.3 ± 3	0.02
GGO, Follow-up	4.4 ± 1	3.8 ± 1	0.1 ± 1	<0.01
Consolidations, acute phase	2.9 ± 2	3.7 ± 2	4.2 ± 2	0.33
Consolidations, Follow-up	0	1.2 ± 1	0.1 ± 1	0.21
Reticulations, acute phase	0	0	0	-
Reticulations, Follow-up	3.5 ± 1	0.6 ± 1	0.2 ± 1	<0.01
Traction bronchiectasis, acute phase	0	0	0	-
Traction bronchiectasis, follow-up	1.9 ± 1	0	0	<0.01

GGO: ground glass opacities.

**Table 3:** Summary of miRNAs and related mRNA targets.

ID	mRNA Target
miR128-3p	TNF-alfa
miR17-5p	NLRP3 *
miR146a-5p	NLRP3
miR21-5p	HIF1-alfa/alfa-SMA/Collagene-1
miR223-3p	NLRP3 *
miR100-5p	mTOR
miR210-3p	ATG7
miR141-3p	NLRP4
miR106a-5p	IL-10/STAT3

* validated interaction. TNF: tumour necrosis factor; NLRP: Nucleotide-binding oligomerization domain, Leucine-rich Repeat, and Pyrin domain containing; HIF: Hypoxia-inducible factor; SMA: smooth muscle actin; mTOR: mechanistic target of rapamycin; ATG7: Autophagy related 7; IL: interleukin; STAT: Signal transducer and activator of transcription.

## Data Availability

The data presented in this study are available on reasonable request from the corresponding author.
